# The Peroxisomal-CoA Synthetase MoPcs60 Is Important for Fatty Acid Metabolism and Infectious Growth of the Rice Blast Fungus

**DOI:** 10.3389/fpls.2021.811041

**Published:** 2022-01-26

**Authors:** Ting Zhang, Ya-Nan Li, Xue Li, Wangliu Gu, Emily Kolojane Moeketsi, Ruiwen Zhou, Xiaobo Zheng, Zhengguang Zhang, Haifeng Zhang

**Affiliations:** Key Laboratory of Integrated Management of Crop Diseases and Pests, Ministry of Education, Department of Plant Pathology, College of Plant Protection, Nanjing Agricultural University, Nanjing, China

**Keywords:** MoPcs60, peroxisome, β-oxidation, fatty acid metabolism, pathogenicity

## Abstract

Fatty acid metabolism is important for the maintenance of fatty acid homeostasis. Free fatty acids, which are toxic in excess, are activated by esterification with coenzyme A (CoA) and then subjected to β-oxidization. Fatty acid β-oxidation-related genes play critical roles in the development and virulence of several phytopathogens. In this study, we identified and characterized a peroxisomal-CoA synthetase in the rice blast fungus *Magnaporthe oryzae*, *MoPCS60*, which is a homolog of *PCS60* in budding yeast. *MoPCS60* was highly expressed during the conidial and early infectious stages and was induced under oleate treatment. Targeted deletion of *MoPCS60* resulted in a significant reduction in growth rate when oleate and olive oil were used as the sole carbon sources. Compared with the wild-type strain Guy11, the Δ*Mopcs60* mutant exhibited fewer peroxisomes, more lipid droplets, and decreased pathogenicity. The distribution of MoPcs60 varied among developmental stages and was mainly localized to peroxisomes in the hyphae, conidia, and appressoria when treated with oleate. Our results suggest that MoPcs60 is a key peroxisomal-CoA synthetase involved in fatty acid β-oxidation and pathogenicity in rice blast fungi.

## Introduction

Fatty acids have various biological functions in cell metabolism and signaling; they are also components of complex membrane lipids and act as precursors for the synthesis of bioactive lipids (Calder, 2015). Fatty acid metabolism is a multistep process critical for maintaining fatty acid homeostasis that involves the breakdown of fatty acids to produce energy. Fatty acid metabolism occurs *via* the well-characterized β-oxidation cycle to produce acetyl-coenzyme A (CoA), which is further metabolized to generate energy and precursors used in other metabolic pathways (Listenberger et al., 2003; van Roermund et al., 2003; Wysham et al., 2016). CoA is a pantothenic acid-containing coenzyme involved in the oxidation of fatty acids in the β-oxidation cycle, and also of pyruvate in the citric acid cycle. Free fatty acids are activated by esterification with CoA and then degraded by β-oxidation. Excess free fatty acids are toxic to cells (Rajvanshi et al., 2017).

Peroxisomes are involved in the degradation of fatty acids, especially long-chain fatty acids (Wang et al., 2007). Peroxisomes are single membrane-bound microbodies that present in all eukaryotic cells. Peroxisomes exert several effects according to environmental cues and metabolic requirements (Koch et al., 2010; Chen et al., 2016; Francisco et al., 2017). Peroxisomes play crucial roles in various catabolic processes including methanol and fatty acid metabolism, among other biosynthetic processes (Yan et al., 2005; Goh et al., 2011; Hagen et al., 2015; Chen et al., 2016; Li L. et al., 2017). Peroxisome biogenesis, matrix protein transport, and peroxisome-associated protein degradation are essential for cellular growth and differentiation (Lingard et al., 2009; Zhong et al., 2016). Peroxisome dysfunction in humans can be classified as deficiencies in a single peroxisomal enzyme, or peroxisomal biogenesis disorders such as Zellweger syndrome, neonatal adrenoleukodystrophy, and infantile Refsum disease (Subramani et al., 2000; Liu et al., 2012; Walter and Erdmann, 2019).

*Magnaporthe oryzae* is the causal agent of rice blast, a devastating disease affecting rice plants worldwide. *M. oryzae* is a filamentous fungus that exhibits high adaptability to external environments and can rapidly mobilize lipids *via* the MAP kinase and cAMP signaling pathways during appressorium development (Wang et al., 2005; Ramos-Pamplona and Naqvi, 2006; Patkar et al., 2012). Long-chain fatty acids are degraded by peroxisomal β-oxidation pathways to yield acetyl-CoA (which is released from triglycerides), glycerol (which is essential for appressorium turgor pressure), and energy (de Jong et al., 1997; Patkar et al., 2012). The organelles and biochemical pathways that break down fatty acids, such as peroxisomal β-oxidation and the glyoxylate cycle, are involved in the regulation of infection-related morphogenesis (Fernandez et al., 2014). Two conserved members of the Zn2-Cys6 family of transcriptional regulators, Far1 and Far2, are essential for lipid droplet mobilization during appressorium development and maturation (bin Yusof et al., 2014). The peroxisomal carnitine acetyl transferase activity protein Pth2, which is induced by acetate and lipids, maintains the acetyl-CoA pools necessary for appressorium function (Bhambra et al., 2006).

Peroxisomal-CoA synthetase 60 (Pcs60) belongs to the family of AMP-binding proteins (Fabian and Ralf, 1996). Pcs60 contains a C-terminal tripeptide peroxisomal targeting signal 1 (PTS1) motif, which is required for targeting peroxisomes *via* Pex5; its expression is induced under oleic acid growth conditions (Fabian and Ralf, 1996; Hagen et al., 2015). Furthermore, Pcs60 is an acyl-activating enzyme that converts oxalate to oxalyl-CoA and regulates responses to biotic and abiotic stresses in plants and yeast (Foster et al., 2012; Foster and Nakata, 2014; Frohlich et al., 2015; Mulleder et al., 2016; Yang et al., 2018). However, the function of Pcs60 proteins in filamentous fungi remains unknown. In this study, we characterized the function of *M. oryzae* Pcs60 (MoPcs60) in rice blast fungus and found that Pcs60 plays an important role in the utilization of long-chain fatty acids, peroxisome biosynthesis, lipid droplet degradation, and invasive hyphal (IH) growth in *M. oryzae*.

## Materials and Methods

### Fungal Strains and Culture Conditions

The *M. oryzae* Guy11 isolate was used as the wild-type strain throughout. The Δ*Mopcs60* mutant and the complemented strains *MoPCS60-C* were generated in this study. For vegetative growth assay, 3 mm × 3 mm agar blocks of the indicated strains were placed onto CM, SDC, and minimal media (MM) supplementing with 1% (w/v) glucose, 2% (w/v) olive, 1 mM sodium butyrate, sodium laurate, and sodium oleate as sole carbon source, respectively. All indicated strains were prior to incubation at 28°C for 7 days and photographed.

### Complementation of the *S. cerevisiae* Δ*Scpcs60* Mutant

The cDNA of *MoPCS60* was amplified using primers pYES2-F/pYES2-R (Supplementary Table 1) and then cloned into the yeast expression vector pYES2 plasmid (Invitrogen). Colonies were cultured on synthetic dropout (SD) medium without uracil. The empty pYES2 vector was transformed into the Δ*Scpcs60* mutant as a control. All strains were diluted to an *OD*_600_ = 0.1, after that 5 μl of 10-fold serial dilutions was cultured on YPG plates containing 6 μg/ml adriamycin (Lum et al., 2004) and 2% galactose at 30°C for 3 days.

### *MoPCS60* Gene Deletion and Complementation

To construct the *MoPCS60* gene knockout vector, approximately 1-kb fragments franking the targeted gene were amplified with primers (Supplementary Table 1). Then, the PCR products were digested with *Sal*I/*Eco*RI and *Bam*HI/*Sac*I and cloned into the pCX62 plasmid. The resulting gene knockout vector was transformed into protoplasts of Guy11. The hygromycin-resistant transformants were screened by PCR and verified by Southern blot (Supplementary Figure 1). To generate the complemented transformants, fragment containing the putative promoter and *MoPCS60* encoding region was amplified and cloned into pYF11 (bleomycin resistance) using the yeast gap repair approach (Bruno et al., 2004). The resulting vector was sequenced and transformed into protoplasts of the Δ*MoPcs60* mutant.

### Quantification of Gene Expression by qRT-PCR

Total RNA samples were extracted from mycelia, conidia, and infected rice leaves using PureLink™ RNA mini kit (Invitrogen) according to the manufacturer’s protocol. Reverse transcriptase HiScript III RT SuperMix for qPCR (Vazyme Biotech Co., Ltd., Nanjing, China) was used to prepare cDNA. qRT-PCR was run on an Applied Biosystems (Foster City, CA, United States) 7500 Real-time PCR System with SYBR Premix Ex Taq (Vazyme Biotech Co., Ltd., Nanjing, China). The 2^–ΔΔ*CT*^ method was used to calculate the relative quantification of each transcript (Livak and Schmittgen, 2001) with the *M. oryzae actin* gene as the internal control. The experiment was repeated three times with three biological replicates. The primers used in this section are listed in Supplementary Table 1.

### Conidiation, Appressorium Formation, and Turgor Analysis

For conidiation, 10-day-old SDC agar cultures were washed with 3 ml H_2_O and counted with a hemocytometer (Zhang et al., 2010). For appressorium formation assays, 25 μl of conidial suspensions (5 × 10^4^ spores/ml) was placed onto a hydrophobic surface (Fisher brand, Germany) and cultured at 28°C. Appressorium formation rate was counted at 24 hpi under a microscope (Zhang et al., 2011; Liu et al., 2018). Appressorium turgor was measured as previously described (Zhong et al., 2016).

### Observation of Lipid Droplet Degradation and Glycogen Translocation During Appressorial Development

Conidial suspensions (25 μl) were incubated on hydrophobic surface. The lipid droplets during appressoria development were stained with Nile red solution as previously described (Zhong et al., 2016). The glycogen during appressorial development was stained with KI solution as previously described (Zhong et al., 2016). The samples were observed under a Zeiss Axio Observer A1 inverted microscope after 3-min incubation.

### Pathogenicity, Aniline Blue, and 3,3′-Diaminobenzidine Staining Assays

For spraying assay, 2-week-old rice seedlings (*Oryza sativa* cv. CO39) were sprayed with 4 ml of conidial suspensions (1 × 10^5^ spores/ml, 0.2% gelatin, w/v) of each strain. The inoculated plants were placed into a moist chamber at 28°C for 24 h and then transferred to another moist chamber under a 12-h light/12-h dark photoperiod for 4 days and photographed (Zhang et al., 2014; Zhong et al., 2016). For detached barley assay, three droplets of conidial suspensions (20 μl, 1 × 10^5^ spores/ml) were drop-inoculated onto 7-day-old detached barley leaves (*Hordeum vulgare cv.* Four-arris) for 5 days and photographed (Zhong et al., 2016). For infectious growth observation, detached rice sheaths were injected with 1 ml of conidial suspensions (1 × 10^5^ spores/ml) and placed into a moist chamber at 28°C. Infectious hyphae (IH) types were examined under a confocal microscope (Zeiss LSM710, 63 × oil) (Liu et al., 2016). Papillary callose deposits were examined by staining with solution (0.067 M K_2_HPO_4_, pH = 9.0) containing 0.05% (w/v) aniline blue (Sigma) at 32 hpi. ROS accumulation was examined by staining with 3,3′-diaminobenzidine (DAB, Sigma-Aldrich, 1 mg/ml) solution (pH = 3.8) for 12 h and destained with a solution (ethanol/acetic acid = 98:2, v/v) for 2 h at 24 hpi.

### Subcellular Localization, Peroxisome, and Lipid Droplet Observation

To investigate the cellular localization, *MoPCS60*-GFP and RFP-Pts1 (a fluorescent marker appended with a type I peroxisomal targeting signal) were constructed and cotransformed into the Δ*Mopcs60* mutants. The resulting transformants were observed under a confocal fluorescence microscope (Zeiss LSM710, 63 × oil) (Zhong et al., 2016). For peroxisome quantity and morphology observation, RFP-Pts1 was transformed into the wild-type Guy11, Δ*Mopcs60* mutant and the complemented strain *MoPCS60-C*, and observed under a confocal fluorescence microscope (Zhong et al., 2016). For lipid droplet staining, vegetative hyphae were cultured in liquid CM for 16 h and then stained with BODIPY™ 493/503 (1 μg/ml) in the dark at 25°C for 3 min (Rajvanshi et al., 2017). Fluorescent images of peroxisomes and lipid droplets were taken under a confocal fluorescence microscope (Zeiss LSM710, 63 × oil).

## Results

### MoPcs60 Is a Homolog of Pcs60 in Budding Yeast

**FIGURE 1:**
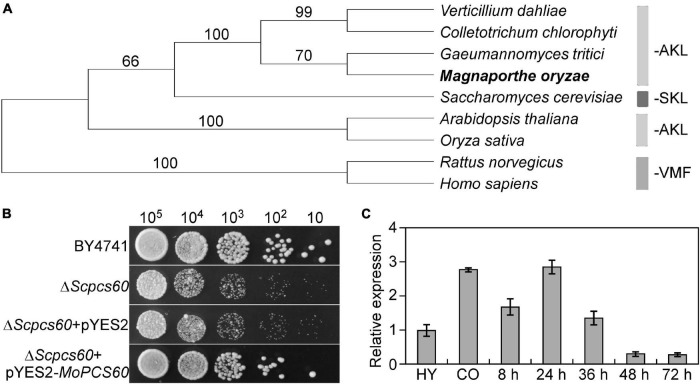
Phylogenetic analysis, yeast complementation, and expression profiles of *MoPCS60*. **(A)** Phylogenetic trees of MoPcs60 proteins from different organisms were constructed using the CLUSTAL_W and MEGA 5.1 programs by the neighbor-joining method with 1000 bootstrap replicates. Species names and GenBank accession numbers are as follows: XP_003712086.1 (*M. oryzae*); OLN86417.1 (*C. chlorophyti*); XP_009223944.1 (*G. tritici R3-111a-1*); XP_009649893.1 (*V. dahliae*); NP_001438.1 (*H. sapiens*); NP_075243.1 (*R. norvegicus*); NP_001054304.1 (*O. sativa*); NP_190468.1 (*A. thaliana*); NP_009781 (*S. cerevisiae*). AKL and SKL: major PTS1 tripeptides, VMF: minor PTS1 tripeptides. The rice blast fungus was showed in bold. **(B)** MoPcs60 partially rescued the growth defect of the yeast Δ*Scpcs60* mutant. Serial dilutions of BY4741, Δ*Scpcs60*, and Δ*Scpcs60* transformed with pYES2 or pYES2-*MoPCS60* were grown on YPG (galactose) with 6 μg/ml adriamycin plates at 30°C for 3 days and then photographed. **(C)** qRT-PCR analyses the expression levels of *MoPCS60* at different fungal developmental stages. RNA was extracted from hyphae cultured for 36 h (HY), conidia (CO), and rice leaves inoculated with the wild-type conidia for 8, 24, 36, 48 and 72 h, respectively. Error bars represent the standard deviations of three replicates.

First, we used the Pcs60 peptide sequence from budding yeast as the query in a BlastP search within the *M. oryzae* genome database in FungiDB^1^. We identified a Pcs60 homolog (MGG_06199) that shared 49% amino acid similarity with Pcs60, which we named MoPcs60. *MoPCS60* encodes a peroxisomal-CoA synthetase comprising 522 amino acids, which contains two conserved AMP-binding domains (Fabian and Ralf, 1996) and a C-terminal PTS1 tripeptide motif required for peroxisomal targeting (Hagen et al., 2015). Our phylogenetic analysis revealed that Pcs60 is well-conserved across various organisms including yeast, filamentous fungi, plants, mammals, and homo sapiens (Figure 1A). Heterologous complementation assays revealed that *MoPCS60* rescued the phenotype of a *Saccharomyces cerevisiae*Δ*pcs60* mutant exposed to adriamycin (Figure 1B), indicating that MoPcs60 is a homolog of yeast Pcs60. Moreover, *MoPCS60* expression was higher during the conidial (2.8-fold) and early infectious (2.9-fold) stages compared with in the mycelial stage (Figure 1C), indicating a potential role of *MoPCS60* in conidiation and infection in rice blast fungus.

### MoPcs60 Is Not Involved in Vegetative Growth, Conidiation, or Appressorium Formation

To investigate the biological function of MoPcs60 in *M. oryzae*, we generated a *MoPCS60* deletion mutant by replacing the *MoPCS60* coding region with the hygromycin phosphotransferase gene, through polyethylene glycol-mediated protoplast transformation. The *MoPCS60* gene deletion in the Δ*Mopcs60* mutant was confirmed by Southern blot analysis (Supplementary Figure 1). The complemented transformant strain *MoPCS60-C* was generated by transforming a genetic construct containing *MoPCS60* fused to green fluorescent protein (*MoPCS60-*GFP) into Δ*Mopcs60* protoplasts. The vegetative growth, conidial production, and appressorium formation phenotypes of Δ*Mopcs60* and the complemented transformant strain *MoPCS60-C* were similar to those of the wild-type strain Guy11 (Table 1), which suggests that MoPcs60 plays no role in vegetative growth, conidiation, or appressorium formation in *M. oryzae*.

**TABLE 1:** Phenotypic analysis of Guy11, Δ*Mopcs60*, and *MoPCS60-C* strains.

Strain	Colony diameter (cm)^a^		Conidiation (×10^4^/cm^2^)^b^	Appressorium formation (%)^c^	Collapsed appressorium (%)^d^			
	CM	SDC			1 M	2 M	3 M	4 M
Guy11	5.5 ± 0.1	4.0 ± 0.1	12.9 ± 0.9	94.5 ± 2.1	48.5 ± 3.5	78.5 ± 2.1	80.5 ± 2.1	94.5 ± 0.7
Δ*Mopcs60*	5.4 ± 0.1	4.0 ± 0.1	11.5 ± 1.2	93.0 ± 2.8	46.0 ± 2.8	76.0 ± 2.8	80.5 ± 0.7	93.5 ± 0.7
*MoPCS60-C*	5.5 ± 0.1	4.0 ± 0.1	12.1 ± 0.7	93.0 ± 1.4	49.5 ± 3.5	78.0 ± 1.4	81.5 ± 2.1	94.0 ± 1.4

*±Standard deviation (±SD) was calculated from three repeated experiments.*

*^a^Colony diameter of the indicated strains on CM and SDC media after 7 days of incubation at 28°C.*

*^b^Quantification of the conidial production of the indicated strains from 10 days of SDC cultures.*

*^c^Appressorium formation on hydrophobic surfaces at 24 h postinoculation (hpi).*

*^d^Percentage of collapsed appressorium under the treatment of 1–4 M glycerol.*

### MoPcs60 Is Important for Infectious Hyphal Growth

**FIGURE 2:**
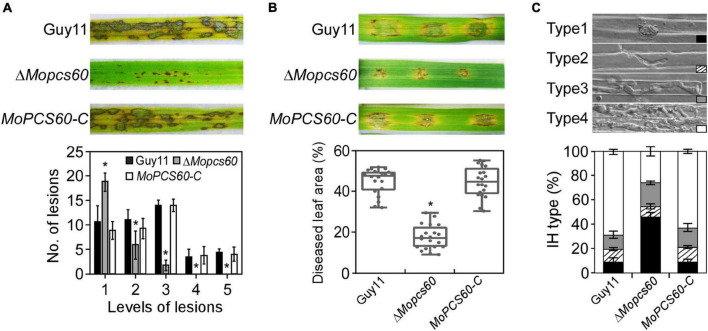
The Δ*Mopcs60* mutant is defective in pathogenicity. **(A)** Conidial suspensions were sprayed onto rice seedlings and examined at 5 dpi (days postinoculation) (Top). Lesion types were counted within an area of 4 cm^2^ leaf from each strain (Bottom). Error bars represent the standard deviations of three replicates; asterisk represents significant difference (*p < 0.01). **(B)** Conidial suspensions were inoculated onto detached barley leaves and examined at 5 dpi (Top). Diseased leaf area (Bottom) was analyzed by ImageJ (Media Cybernetics Inc., Shanghai, China). The percentage was represented by a box-dot-plot. Statistical analysis was performed using GraphPad Prism 8.0.1, and asterisk represents significant difference (*p < 0.01). **(C)** Detached rice sheaths were injected with conidial suspensions. IH types were counted and statistically analyzed at 32 hpi. Error bars represent the standard deviations of three replicates.

To examine the role of MoPcs60 in fungal pathogenicity, conidial suspensions of Guy11, Δ*Mopcs60*, and *MoPCS60-C* were sprayed onto susceptible rice seedlings. After 5 days of incubation, numerous typical lesions had formed on rice leaves inoculated with Guy11 and *MoPCS60-C*. By contrast, many smaller, restricted lesions had formed on rice leaves inoculated with the Δ*Mopcs60* mutant (Figure 2A). The lesions on the rice leaves were graded based on a “lesion-type” assay (Liu et al., 2018). We found that the Δ*Mopcs60* mutant formed more type 1, and fewer type 2 and type 3 lesions compared with Guy11 and *MoPCS60-C* (Figure 2A); no type 4 or type 5 lesions were present on leaves inoculated with Δ*Mopcs60*. We then performed a barley infection assay, which showed that the pathogenicity was reduced in Δ*Mopcs60* compared with Guy11 and *MoPCS60-C*, as shown by the smaller lesions (Figure 2B). Furthermore, we examined the penetration and IH growth of Δ*Mopcs60*, Guy11, and *MoPCS60-C* in rice sheath cells. More than 60% of the rice cells showed type 4 IH growth when inoculated with Guy11 and *MoPCS60-C* conidia, whereas over 70% of rice cells showed type 1, type 2, or type 3 IH growth when inoculated with Δ*Mopcs60* conidia (Figure 2C). In addition, we examined endogenous reactive oxygen species (ROS) production in infected cells and found that there was no significant difference in ROS production following infection with Guy11 or Δ*Mopcs60* (Supplementary Figures 2A,B). These results suggest that MoPcs60 plays an important role in infectious hyphal growth.

### The Pathogenicity of Δ*Mopcs60* Is Not Due to Appressorium Turgor or Penetration

Appressorium-mediated penetration of *M. oryzae* is a turgor-driven process associated with substantial glycerol accumulation in the infected cell (Ramos-Pamplona and Naqvi, 2006). To investigate whether the reduced pathogenicity of Δ*Mopcs60* was associated with appressorium-mediated penetration, we first determined the rate of appressorium collapse in the presence of various hyperosmotic concentrations of glycerol by performing an incipient cytorrhysis assay. We found that there was no significant difference in appressorium turgor between Δ*Mopcs60* and Guy11 (Table 1), which suggests that MoPcs60 is not necessary for the generation or maintenance of appressorium turgor.

Glycogen translocation and lipid degradation are required for appressorial maturation and appressorium-mediated host penetration (Wang et al., 2005; Zhong et al., 2016; Li X. et al., 2017). Therefore, we examined the cellular degradation of lipid droplets during appressorium development by Nile red staining. Lipid droplets were gradually degraded during appressorial maturation, and there was no difference in intracellular lipid storage between Δ*Mopcs60* and Guy11 (Supplementary Figure 3A). Moreover, we examined glycogen storage by iodine solution staining and found that glycogen translocation was not significantly different between Δ*Mopcs60* mutant and Guy11 (Supplementary Figure 3B). These results indicated that MoPcs60 is not necessary for lipid droplet degradation or glycogen translocation in the appressorium.

**FIGURE 3:**
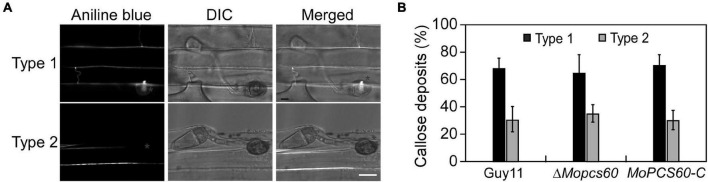
Papillary callose deposits are not changed in the Δ*Mopcs60* mutant. **(A)** Aniline blue staining to visualize appressorium of the wild-type Guy11, Δ*Mopcs60* mutant, and the complemented transformant *MoPCS60-C* at 32 hpi on rice leaves. Bar = 5 μm. **(B)** Statistically analyzed the number of papillary callose deposits formed underneath appressoria. Error bars represent the standard deviations of three replicates.

Next, we evaluated the penetration pegs using aniline blue staining, which stains papillary callose deposits (Sun et al., 2006; Shi et al., 2021). The callose deposits formed by Δ*Mopcs60* were not significantly different to those of Guy11 at 32 h postinoculation (Figures 3A,B). These results indicated that the pathogenicity of Δ*Mopcs60* was not associated with appressorium turgor or penetration.

### MoPcs60 Is Involved in the Utilization of Long-Chain Fatty Acids

**FIGURE 4:**
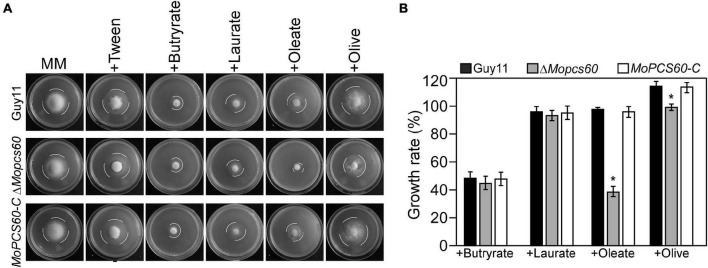
The Δ*Mopcs60* mutant shows defect in utilization of long-chain fatty acids. **(A)** The wild-type strain Guy11, Δ*Mopcs60* mutant, and the complemented transformant *MoPCS60-C* were grown on minimal medium and MM with butyrate (1 mM), laurate (1 mM), olive (1% w/v), and oleate (1 mM) as the sole carbon sources and incubated at 28°C for 7 days. **(B)** Statistical analysis of the growth rate of the indicated strains on different media. Error bars are standard deviations from three biological repeats, and asterisk represents significant differences at *p < 0.01.

To investigate the role of MoPcs60 in fatty acid metabolism, we inoculated minimal medium plates containing short-chain (butyrate), medium-chain (laurate), or long-chain (oleate and olive oil) fatty acids as the sole carbon source with Guy11, Δ*Mopcs60*, or *MoPCS60-C*. After 7 days of incubation, the growth rate of the Δ*Mopcs60* mutant was significantly lower on oleate and olive oil plates compared with Guy11 and *MoPCS60-C*. There was no significant difference in growth rate among the three tested strains on butyrate or laurate plates (Figures 4A,B). Our results suggested that MoPcs60 is involved in long-chain fatty acid utilization.

### *MoPCS60* Deletion Reduces Peroxisome Quantity and Increases the Number of Lipid Droplets

**FIGURE 5:**
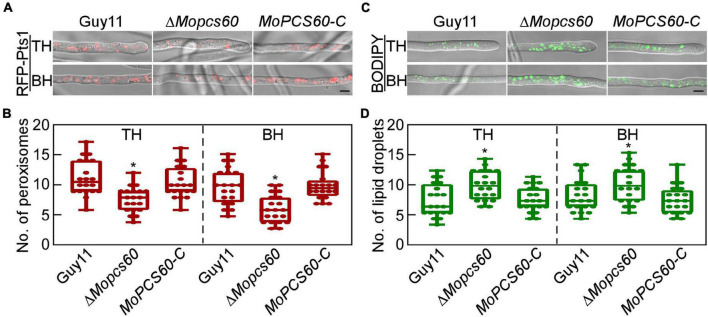
The Δ*MoPcs60* mutant shows a decreased number of peroxisomes and an increased number of lipid droplets. **(A)** Peroxisomes were examined in tip (TH) and basal (BH) hyphae of Guy11, Δ*Mopcs60*, and *MoPCS60-C* strains expressing RFP-Pts1 protein. Differential interference contrast (DIC) and fluorescent images were taken under a confocal microscope. Bar = 5 μm. **(B)** Statistically analyzed the number of peroxisomes in 1 × 10^3^ μm from 20 fields of view in each strain and represented by a box-dot-plot. Statistical analysis was performed using GraphPad Prism 8.0.1, and asterisks represent significant differences at *p < 0.01. **(C)** Lipid droplets were examined in TH and BH of Guy11, Δ*Mopcs60*, and *MoPCS60-C* strains stained by BODIPY 493/503. Bar = 5 μm. **(D)** Statistically analyzed the number of lipid droplets in 1 × 10^3^ μm from 20 fields of view in each strain and represented by a box-dot-plot. Statistical analysis was performed using GraphPad Prism 8.0.1, and asterisks represent significant differences at *p < 0.01.

Peroxisomes are ubiquitous organelles intimately involved in fatty acid metabolism (Yan et al., 2005; Wang et al., 2007). To investigate whether the dysfunctional fatty acid metabolism in Δ*Mopcs60* was related to peroxisomes, we examined the morphology and quantity of peroxisomes in Guy11, Δ*Mopcs60*, and *MoPCS60-C*, all of which expressed red fluorescent protein (RFP) fused to a “PTS1 targeting peptide” (RFP-Pts1). We found that the number of peroxisomes was markedly lower in the Δ*Mopcs60* mutant compared with the other two strains (Figures 5A,B). Defective fatty acid metabolism causes an increase in non-polar lipid content (Rajvanshi et al., 2017). Therefore, we quantified the lipid droplets and examined their morphology in Guy11, Δ*Mopcs60*, and *MoPCS60-C* by staining with BODIPY™ 493/503; the number of droplets was significantly higher in the Δ*Mopcs60* mutant than the other two strains (Figures 5C,D). However, the morphologies of the peroxisomes and lipid droplets were similar among all three strains examined (Figures 5A,C). These results suggest that MoPcs60 plays a critical role in peroxisome biogenesis and lipid droplet degradation in rice blast fungus.

### MoPcs60 Localizes to Peroxisomes Under Oleate Induction

**FIGURE 6:**
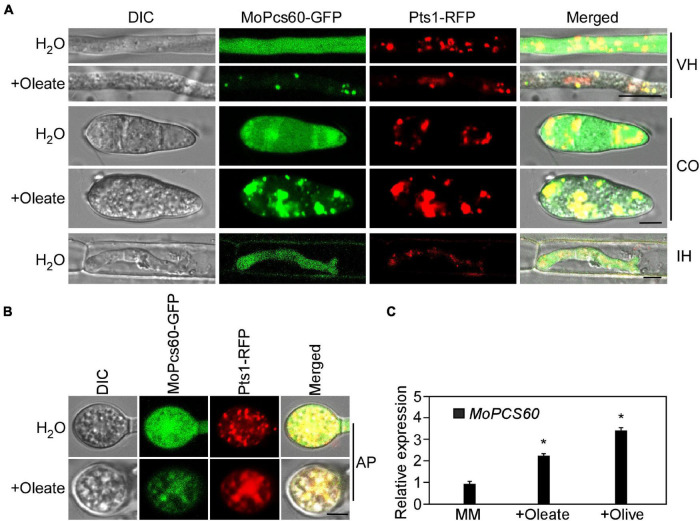
MoPcs60 localizes to peroxisomes under oleate induction. **(A,B)** Hypha, conidium, invasive hyphae, and appressorium of the *MoPCS60-C* strain coexpressing *MoPCS60*-GFP and RFP-Pts1 with or without oleate treatment were observed under a confocal microscope. VH, vegetative hyphae; CO, conidium; IH, invasive hyphae; AP, appressorium. Bar = 5 μm. **(C)** qRT-PCR analyses the expression levels of *MoPCS60* in wild-type Guy11 with or without oleate and olive treatment. Error bars are standard deviations from three biological repeats, and asterisk represents significant difference (*p < 0.01).

To investigate the subcellular localization of MoPcs60, we generated a Δ*Mopcs60* mutant strain that coexpressed MoPcs60-GFP and RFP-Pts1, which we examined using confocal microscopy. MoPcs60-GFP was distributed throughout the cells, being present in the hyphae, conidium, appressorium, and IH (Figures 6A,B). Due to the involvement of MoPcs60 in oleate metabolism, we examined the localization of MoPcs60-GFP following oleate treatment. We found that MoPcs60-GFP was mainly distributed in the punctate structures, which colocalized with RFP-Pts1 in the hyphae, conidium, and appressorium under oleate treatment (Figures 6A,B). In addition, we analyzed the expression levels of *MoPCS60* under oleate and olive oil treatment and found that *MoPCS60* expression was greater than 2-fold higher under the long-chain fatty acid treatment (Figure 6C). These results indicated that the expression and localization of MoPcs60 were related to oleate metabolism in *M. oryzae*.

## Discussion

In this study, we identified and characterized the peroxisomal-CoA synthetase MoPcs60 in *M. oryzae* and found that it plays an important role in long-chain fatty acid utilization, peroxisome biosynthesis, lipid droplet degradation, and invasive growth. Our findings demonstrated that fatty acid metabolism mediated by MoPcs60 is critical for the development and pathogenicity of *M. oryzae*, and provide new insight into the fundamental molecular relationship between fatty acid metabolism and phytopathogen pathogenicity.

Free fatty acids are activated by esterification with CoA and then oxidized and metabolized (Daum et al., 2007). Fatty acid metabolism is critical for the maintenance of fatty acid and energy homeostasis, and excess free fatty acids are toxic (Carballeira, 2008; Rajvanshi et al., 2017). Indeed, fungal strains with dysfunctional fatty acid metabolism exhibit growth, peroxisome biogenesis, and mitochondrial defects when grown on fatty acid-supplemented media. In *M. oryzae*, an *MoMFP1* (*FOX2*, a multifunctional enzyme) deletion prevented the utilization of fatty acids as the sole carbon source and attenuated *M. oryzae* virulence (Wang et al., 2007). Furthermore, the fatty acid synthase β*-*subunit dehydratase Fas1 is essential for peroxisome biogenesis, β-oxidation, and pathogenesis (Sangappillai and Nadarajah, 2020). Consistent with these findings, our data showed that deletion of *MoPCS60* led to defects in the utilization of long-chain fatty acids, peroxisome biogenesis, and infectious growth in *M. oryzae*. Moreover, lipid catabolism genes are highly expressed during the early stages of infection in the phytopathogen *Blumeria graminis* (Both et al., 2005). Taken together, these findings suggest that fatty acid metabolism is important for fungal pathogenesis.

In *M. oryzae*, lipid droplets move to the appressorium following conidial germination. Lipid droplet degradation is associated with glycerol accumulation, which is required for generating turgor pressure during penetration (Wang et al., 2007; Sangappillai and Nadarajah, 2020). Although the Δ*Mopcs60* mutant exhibited defects in fatty acid metabolism and virulence, the mobilization and breakdown of lipid droplets during turgor generation were not impaired in the appressorium; this is in contrast to the mutants Δ*Momfp1*, Δ*far1*, Δ*far2*, and Δ*far1*Δ*far2* (Wang et al., 2007; bin Yusof et al., 2014). This indicates that MoPcs60 is not involved in lipid droplet mobilization during appressorium development, and that MoPcs60 is not essential for appressorium-mediated infection in *M. oryzae*. In addition, there were no apparent differences in callose deposits or ROS accumulation between the wild-type and mutant strains, indicating that MoPcs60 was not involved in penetration peg formation or host immunity suppression. Therefore, MoPcs60 deletion likely disrupted CoA synthesis and peroxisome biogenesis, which in turn prevented the oxidation of free fatty acids, causing fatty acid toxicity in IH.

Peroxisomal-CoA synthetase 60 is a PTS1-containing protein that can be induced under oleic acid growth conditions in *S. cerevisiae* (Fabian and Ralf, 1996). In contrast to the yeast Pcs60, which is constitutively expressed and localized in peroxisomes, MoPcs60 should be treated with oleate and is distributed outside of peroxisomes. This disparity in expression and localization suggests that the function of Pcs60 differs between *S. cerevisiae* and *M. oryzae*. In addition, *MoPCS60* expression levels were high under oleate treatment and were comparable to the *MoPCS60* expression levels observed during the early stage of infection. Therefore, MoPcs60 might play a role in the response to host fatty acids during infectious growth. However, MoPcs60 is mainly distributed in the cytosol of IH, which suggests that there are unknown MoPcs60-related regulatory mechanisms at play during infection. MoPcs60 functions as an acyl-activating enzyme involved in oxalic acid metabolism in yeast and plants (Foster et al., 2012; Foster and Nakata, 2014; Xian et al., 2020); such potential roles for MoPcs60 should be investigated in *M. oryzae*.

## Conclusion

We demonstrated that the peroxisomal-CoA synthetase MoPcs60 plays a critical role in fatty acid metabolism, peroxisome biosynthesis, lipid droplet degradation, and invasive growth in *M. oryzae*. MoPcs60 mainly localizes to peroxisomes in the hyphae, conidia, and appressorium in response to extracellular oleate. These results provide new insight into the pathogenic mechanisms of *M. oryzae*.

## Data Availability Statement

The datasets presented in this study can be found in online repositories. The names of the repository/repositories and accession number(s) can be found in the article/Supplementary Material.

## Author Contributions

TZ, ZZ, and HZ designed the research and wrote the manuscript. TZ, YL, XL, WG, EM, and RZ performed the experiments. TZ, HZ, and XZ analyzed the data. All authors contributed to the article and approved the submitted version.

## Conflict of Interest

The authors declare that the research was conducted in the absence of any commercial or financial relationships that could be construed as a potential conflict of interest.

## Publisher’s Note

All claims expressed in this article are solely those of the authors and do not necessarily represent those of their affiliated organizations, or those of the publisher, the editors and the reviewers. Any product that may be evaluated in this article, or claim that may be made by its manufacturer, is not guaranteed or endorsed by the publisher.

## Footnotes

1
https://fungidb.org/fungidb/app

## Supplementary Material

Supplementary Figure 1 Targeted deletion of *MoPCS60* in *M. oryzae*. **(A)** *MoPCS60* gene replacement strategy in *M. oryzae* genome. **(B)** *MoPCS60* gene knockout mutants were verified by Southern blot analysis with probe 1 (gene fragment) and probe 2 (*HPH*), respectively. The genomic DNA was digested with *Eco*RI.

Supplementary Figure 2 MoPcs60 is not involved in scavenging host-derived ROS. **(A)** DAB staining to visualize infection hyphae of the wild-type Guy11, Δ*Mopcs60* mutant, and the complemented transformant *MoPCS60-C* at 24 hpi in infected cells. Bar = 100 μm. **(B)** Number of no reddish-brown precipitate in infected cells was counted and analyzed. Error bars represent the standard deviations of the three replicates.

Supplementary Figure 3 MoPcs60 is not necessary for lipid droplets degradation and glycogen translocation during appressorium development. **(A)** Lipid droplets of conidia and appressoria were stained by Nile red at different time points and observed under a fluorescence microscope. Error bars represent the standard deviations of three replicates. Bar = 20 μm. **(B)** Glycogen deposits of conidia and appressoria were stained by iodine solution at different time points and observed under a fluorescence microscope. Error bars represent the standard deviations of three replicates. Bar = 20 μm.
