# Supplementary material for: The Peroxisomal-CoA Synthetase MoPcs60 Is Important for Fatty Acid Metabolism and Infectious Growth of the Rice Blast Fungus
Source: Front Plant Sci. 2022 Jan 26;12:811041. doi: 10.3389/fpls.2021.811041 (PMC8826238; doi:10.3389/fpls.2021.811041)
Supplement: Supplementary file 1 [file Table_1.DOCX]

Table S1. Primers used in this study

| Primer name | Sequence (5’-3’) | Remark |
| --- | --- | --- |
| pYES2-F | TTAAGCTTGGTACCGAGCTCATGGCTGCGCCAAACACTCT | amplification of *MoPCS60* coding sequence |
| pYES2-R | TACATGATGCGGCCCTCTAGACAGCTTAGCCTTGTTGTCTT | amplification of *MoPCS60* coding sequence |
| *PCS60*-F1 | ACGCGTCGACATCTCATGACAGCGGGATGACT | amplification of *MoPCS60* 5’ flanking sequence |
| *PCS60*-R2 | CCGGAATTCAGAGTGTTTGGCGCAGCCAT | amplification of *MoPCS60* 5’ flanking sequence |
| *PCS60*-F3 | CGCGGATCCGCTGTGAGATCAATATGTG | amplification of *MoPCS60* 3’ flanking sequence |
| *PCS60*-R4 | CGAGCTCTCATGGTGATGACGCAAGG | amplification of *MoPCS60* 3’ flanking sequence |
| *PCS60*koin-F | ACGAGCGGCTACCTCAAC | validation of *MoPCS60* deletion transformants |
| *PCS60*koin-R | TCGTCTCGGCGACTATGC | validation of *MoPCS60* deletion transformants |
| Pro-*PCS60*-F | CACCACCCCGGTGAACAGCTCCTCGCCCTTGCTCACCAGCTTAGCCTTGTTGTCTT | *MoPCS60* complementation |
| Pro-*PCS60*-R | TTTCGTAGGAACCCAATCTTCAAAATGGCTGCGCCAAACACTCTCAA | *MoPCS60* complementation |
| Q*PCS60*-F | TCGTCATGCCGCTCTTCCAC | qRT-PCR analysis of *MoPCS60* |
| Q*PCS60*-R | TCGTTACCATCGCCGTCCAG | qRT-PCR analysis of *MoPCS60* |
