# Supplementary figures and images for: The Peroxisomal-CoA Synthetase MoPcs60 Is Important for Fatty Acid Metabolism and Infectious Growth of the Rice Blast Fungus
Source: Front Plant Sci. 2022 Jan 26;12:811041. doi: 10.3389/fpls.2021.811041 (PMC8826238; doi:10.3389/fpls.2021.811041)

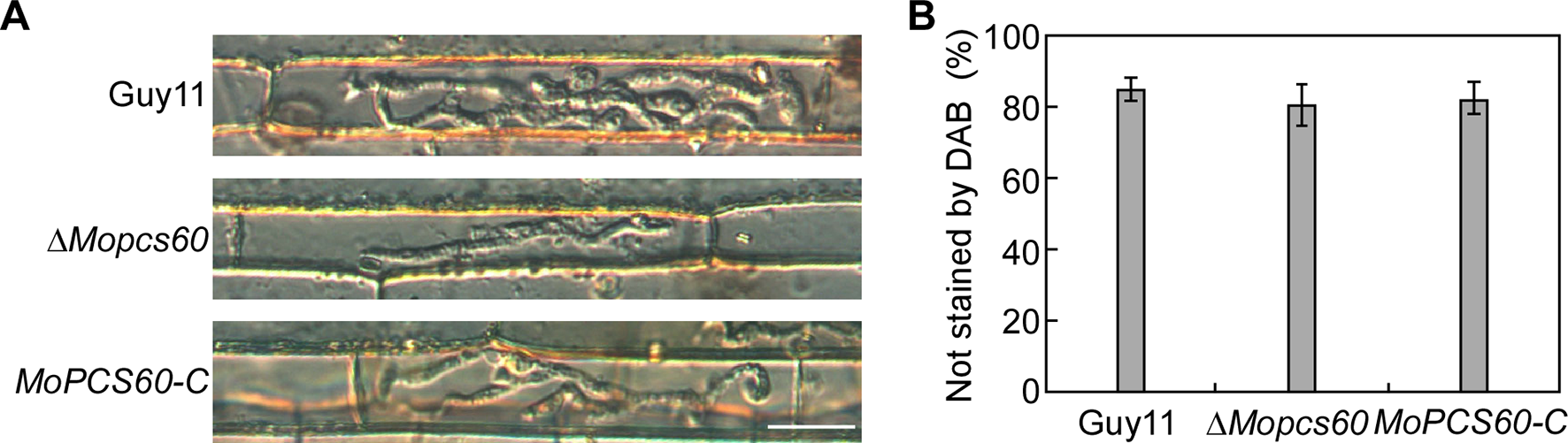

Supplement: Supplementary Figure 2 — MoPcs60 is not involved in scavenging host-derived ROS. (A) DAB staining to visualize infection hyphae of the wild-type Guy11, ΔMopcs60 mutant, and the complemented transformant MoPCS60-C at 24 hpi in infected cells. Bar = 100 μm. (B) Number of no reddish-brown precipitate in infected cells was counted and analyzed. Error bars represent the standard deviations of the three replicates. [file Image_2.TIF]

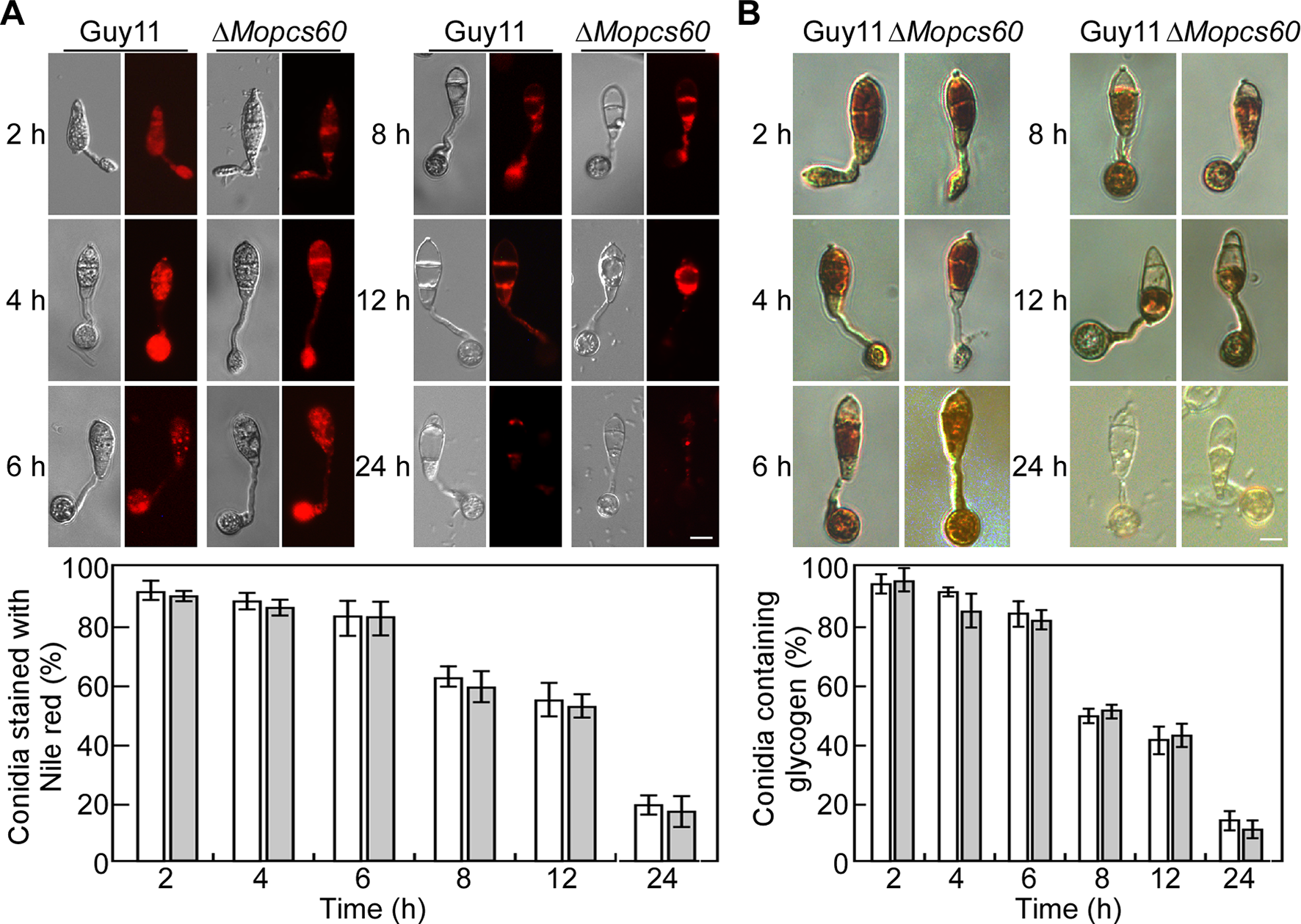

Supplement: Supplementary Figure 3 — MoPcs60 is not necessary for lipid droplets degradation and glycogen translocation during appressorium development. (A) Lipid droplets of conidia and appressoria were stained by Nile red at different time points and observed under a fluorescence microscope. Error bars represent the standard deviations of three replicates. Bar = 20 μm. (B) Glycogen deposits of conidia and appressoria were stained by iodine solution at different time points and observed under a fluorescence microscope. Error bars represent the standard deviations of three replicates. Bar = 20 μm. [file Image_3.TIF]
